# Preliminary Experience of Robotic Endometriosis From a Tertiary Care Center in India: The Learning Curve

**DOI:** 10.7759/cureus.104335

**Published:** 2026-02-26

**Authors:** Anitha Kunnaiah, Keerthana M

**Affiliations:** 1 Gynaecology and Obstetrics, Robotic Sciences, Yashoda Hospitals, Hyderabad, IND

**Keywords:** deep infiltrating endometriosis, endometriosis, learning curve, pain scores, quality of life scores (ehp-30), robotic-assisted surgery

## Abstract

Background

Endometriosis often requires surgery, especially in deep-infiltrating cases unresponsive to medical therapy. This study presents our institutional experience with robotic-assisted surgery (RAS) for endometriosis in Indian patients.

Methodology

This retrospective study was conducted at a high-volume tertiary care center in India and included patients who underwent robotic-assisted endometriosis surgeries using the da Vinci Xi system between October 2023 and April 2025.

Results

This study analyzed 108 patients, with 39.8% diagnosed with deep infiltrating endometriosis (DIE). The mean age and body mass index of the study groups were 40.9 ± 6.6 years and 26.3 ± 3.3 kg/m², respectively. The average operative time was 129.8 ± 56.0 minutes, with minimal intraoperative and postoperative complications (0.9% each), no conversions to open surgery, no wound infections, and no re-operations. Subgroup analysis revealed that DIE cases had longer operative times. Though complication rates and post-anesthesia care unit stay remained comparable to the non-DIE subgroup. Both subgroups demonstrated significant improvements in postoperative pain and quality of life scores (p < 0.001) compared to their preoperative values. The risk-adjusted cumulative sum analysis of operative and docking times demonstrated distinct learning phases, with marked improvements in surgical efficiency and operating room workflow over time. Notably, a marked inflection point was observed around case 28 in the DIE subgroup. While this subgroup may represent a particularly suitable cohort for the application of robotic techniques, these findings should be interpreted as reflective of procedural optimization within a carefully selected patient population rather than as a definitive statement of superiority.

Conclusions

This study confirms the feasibility, safety, and effectiveness of RAS for endometriosis in India, with significant postoperative improvements and low complication rates across all stages, including complex DIE cases.

## Introduction

Endometriosis is characterized by the presence of endometrial glands and stroma outside the uterine cavity. Globally, it affects approximately 10% of women of reproductive age [1]. The condition is broadly classified into three main types: superficial (peritoneal), ovarian (endometrioma), and deep infiltrating endometriosis (DIE) [2]. Clinical manifestations vary depending on the location and extent of tissue involvement. Gynecological symptoms commonly include chronic pelvic pain, dyspareunia, and infertility, while gastrointestinal and genitourinary involvement may lead to respective bowel and urinary symptoms [3]. Endometriosis is also a significant contributing factor to female infertility, with reported prevalence ranging from 20% to 50% [4].

Surgical intervention remains a key treatment modality for patients with endometriosis who do not respond adequately to pharmacologic endocrine therapy and for those diagnosed with DIE [5,6]. Laparoscopic surgery is currently considered the gold standard for surgical management, offering reduced morbidity, shorter hospital stays, and faster recovery compared to the conventional open approach [7]. Within the realm of minimally invasive surgery (MIS), robotic-assisted surgery (RAS) has emerged as a promising alternative to laparoscopy. RAS provides enhanced three-dimensional visualization, superior precision in dissection, and greater instrument dexterity through wrist articulation, advantages that may be particularly beneficial in complex DIE cases [8]. A recent international multicenter cross-sectional survey conducted under the auspices of the Scientific Endometriosis Foundation and the European Endometriosis League (EEL) gathered responses from 64 centers across Austria, the Czech Republic, Germany, and Switzerland. The survey explored the use of RAS in endometriosis, including its indications, benefits, challenges, technical considerations, and training aspects. Participating centers reported multiple technical and surgical advantages of RAS, particularly in the management of DIE, where improved outcomes were noted [9]. In the Indian context, the adoption of RAS for benign gynecological conditions has shown a positive upward trend in recent years [10]. However, specific data on the use of RAS for endometriosis within the Indian healthcare system remains lacking. Through this study, we aim to share our institutional experience with RAS for endometriosis in Indian patients, encompassing both superficial and deep infiltrating forms of the disease.

## Materials and methods

The study was conducted in the Department of Gynecology at a tertiary care center in Hyderabad, India. A retrospective review of medical records was performed for all endometriosis cases managed using RAS since the initiation of the hospital’s robotic surgery program. The data collection period spanned from October 2023 to April 2025. The study received approval from the Institutional Ethics Committee (approval number: CC/PP-05/2025) and was conducted following the latest version (2024) of the Declaration of Helsinki and Good Clinical Practice guidelines.

All robotic procedures were performed using the da Vinci Xi Surgical System (Intuitive Surgical, Sunnyvale, CA, USA) by a single experienced surgeon. Data were retrospectively collected from patient medical records and included a comprehensive set of variables. Preoperative parameters recorded were age, sex, body mass index (BMI), parity, presenting symptoms, presence of infertility, history of previous abdominopelvic surgeries, preoperative pain, and quality of life (QoL) scores. Perioperative and postoperative parameters included operative time, estimated blood loss, need for blood transfusion, conversion to open surgery, postoperative complications, wound infections, length of post-anesthesia care unit (PACU) stay, total hospital stay, postoperative pain, QoL scores at various time points, duration of analgesic use, time to ambulation, time to resume daily activities, and time to return to work. Pain was assessed using a Visual Analog Scale (VAS) ranging from 1 to 10. QoL was measured using the Endometriosis Health Profile (EHP-30), a validated tool designed to evaluate patient-reported outcomes related to medical and surgical interventions for endometriosis [11].

Categorical variables were summarized by frequency (%). The chi-square or Fisher’s exact test, as appropriate, was used to determine their association with the group variable (i.e., DIE/non-DIE). Quantitative variables were assessed for approximate normality using the Shapiro-Wilk test. As all the variables were following a normal distribution, these were summarized as mean (SD). Paired t-test was used to compare within-group pre-post values for pain scores and QoL. Student’s t-test was used to compare difference in mean values between the two groups (DIE/non-DIE). All tests used were two-sided. In this study, a p-value less than 0.05 was considered statistically significant. Stata 16.0 statistical software (StataCorp., College Station, TX, USA) was used for data analysis. The surgical learning curve was assessed using the cumulative sum (CUSUM) method, which allows for sequential analysis of cases based on surrogate markers such as operative time, applied in our study. For each surgery, the CUSUM value was calculated chronologically by summing the differences between the individual operative time and the mean operative time across all cases. The CUSUM value for the first case represented the difference between its operative time and the overall mean. For the second case, the CUSUM value was obtained by adding the difference in operative time for that case to the CUSUM value of the first case. This iterative approach was applied across all cases, generating a continuous series of CUSUM values that reflected temporal changes in operative performance.

## Results

Baseline and preoperative variables: overall population

Between October 2023 and April 2025, a total of 108 patients underwent RAS for endometriosis, all of whom were included in the analysis. The mean age of the study population was 40.9 ± 6.6 years, and the mean BMI was 26.3 ± 3.3 kg/m². Approximately 25% of the patients presented with infertility. Based on disease classification, around 60% of cases were diagnosed with Stage I or II endometriosis (peritoneal or ovarian), while 39.8% had DIE (Table 1).

**Table 1:** Descriptive characteristics of the study population (N = 108). SD: standard deviation; BMI: body mass index; DIE: deep infiltrating endometriosis; VAS: Visual Analog Scale; QoL: quality of life; EHP-30: Endometriosis Health Profile questionnaire

Variable	N = 108
Age, mean ± SD, years	40.9 ± 6.6
BMI, mean ± SD, kg/m^2^	26.3 ± 3.3
Parity, mean ± SD	1.3 ± 1.0
Prior abdominal surgeries, including obstetric surgeries, n (%)	65 (60.2)
Infertility, n (%)	28 (25.9)
Stage, n (%)	
Stage 1 and 2	65 (60.2)
Stage 3 and 4 (DIE), n (%)	43 (39.8)
Pain score (VAS scale), preoperative, mean ± SD	7.9 ± 1.1
QoL (EHP-30), preoperative, mean ± SD	78.3 ± 8.3

Perioperative outcomes: overall population

Table 2 presents a detailed overview of the perioperative outcomes for the overall study population.

**Table 2:** Perioperative outcomes of the study population (N = 108). SD: standard deviation; PACU: post-anesthesia care unit

Variable	N = 108
Operative time, mean ± SD, minutes	129.8 ± 56.0
Docking time for the robot, mean ± SD, minutes	5.9 ± 1.3
Surgical disciplines involved, n (%)	
1 (gynecologist only)	98 (90.7)
2 or more than 2 (gynecologist + urologist or colorectal surgeon)	10 (9.3)
Conservative surgery, n (%)	11 (10.2)
Conception within 12 months	2 (18.2)
Were the lesions identified intraoperatively using the da Vinci Xi consistent with preoperative imaging findings?	
Yes, n (%)	108 (100)
No, n (%)	0 (0.0)
Firefly used, n (%)	13 (12.1)
Blood loss, mean ± SD, mL	53.0 ± 47.3
Blood transfusion, n (%)	4 (3.7)
Intraoperative complications, n (%)	1 (0.9)
Conversion to open, n (%)	0 (0.0)
Length of hospital stay, mean ± SD, days	2.2 ± 0.7
PACU stay, mean ± SD, hours	4.0 ± 0.3
Analgesia administration, mean ± SD, days	1.3 ± 0.5
Wound infection, n (%)	0 (0.0)
Postoperative complications (immediate postoperative period), n (%)	1 (0.9)
Postoperative complications (late postoperative period), n (%)	1 (0.9)
Re-operation, n (%)	0 (0.0)
Time to return to activities of daily living, mean ± SD, days	2.2 ± 0.9
Time to ambulation, mean ± SD, hours	11.7 ± 3.1

The mean operative time was 129.8 ± 56.0 minutes, and the average estimated blood loss was 53 mL. Intraoperative complications were minimal, with only one (0.9%) event reported, classified as Clavien-Dindo Grade 3. Similarly, postoperative complications were also limited to one (0.9%) case. The average duration of stay in the PACU was 4.0 hours, and the mean hospital stay was 2.2 days. Most surgeries (90.7%) were performed solely by the gynecology team, with multidisciplinary involvement required in only 10 cases.

Subgroup analysis: deep infiltrating endometriosis cases

A subgroup analysis was performed for patients with DIE classified as Stage III or IV. Baseline characteristics of this subgroup are summarized in Table 3.

**Table 3:** Baseline demographics and disease characteristics of the DIE subgroup (N = 43). SD: standard deviation; BMI: body mass index; DIE: deep infiltrating endometriosis; VAS: Visual Analog Scale; QoL: quality of life; EHP-30: Endometriosis Health Profile questionnaire

Variable	N = 43
Age, mean ± SD, year	36.4 ± 6.2
BMI, mean ± SD, kg/m^2^	26.2 ± 3.1
Parity, mean ± SD	0.6 ± 0.7
Prior abdominal surgeries, including obstetric surgeries, n (%)	24 (55.8)
Infertility, n (%)	22 (51.2)
Pain score (VAS scale), preoperative, mean ± SD	8.4 ± 1.2
QoL (EHP-30), preoperative, mean ± SD	82.2 ± 8.0

The mean age was 36.4 years, and the mean BMI was 26.2 kg/m². Notably, infertility was present in 51.2% of these cases. We compared the perioperative and functional outcomes of the non-DIE subgroup (n = 65) with those of DIE cases (n = 43), as detailed in Table 4.

**Table 4:** Comparison of perioperative and functional outcomes (non-DIE vs. DIE subgroup). *: Indicates there is statistical significance between the two groups; ^#^: p-value calculated using a two-sample t test; ^¥^: p-value calculated using the proportion test. DIE: deep infiltrating endometriosis; SD: standard deviation; PACU: post-anesthesia care unit

Parameters	Non-DIE subgroup (N = 65)	DIE subgroup (N = 43)	t-score/chi- square	P-value
Operative time, mean ± SD, minutes^#^	108.8 ± 49.5	161.4 ± 51.2	5.3267	<0.001*
Docking time for the robot, mean ± SD, minutes^#^	5.9 ± 1.3	5.9 ± 1.4	0.0322	0.9743
Need for blood transfusion (intraoperative), n (%)^¥^	0 (0.0)	4 (9.30)	6.2791	0.023*
Intraoperative complications, n (%)^¥^	0 (0.0)	1 (2.33)	1.5258	0.398
Postoperative complications, n (%)^¥^	1 (1.54)	0 (0.0)	0.6677	>0.99
PACU stay, mean ± SD, hours^#^	4.0 ± 0.0	4.1 ± 0.4	1.7858	0.0770
Length of hospital stay, mean ± SD, days^#^	2.0 ± 0.6	2.6 ± 0.7	4.4271	<0.001*
Time to ambulation, mean ± SD, hours^#^	10.8 ± 2.4	13.1 ± 3.5	4.0511	<0.001*
Time to return to work, mean ± SD, days^#^	3.1 ± 1.2	3.0 ± 1.0	-0.4722	0.6378

The operative time was significantly longer in the DIE group (p < 0.001), and blood loss was also notably higher (p = 0.0089). Further, the need for blood transfusions differed significantly between the two groups (p = 0.023). There were no significant differences in intraoperative or postoperative complication rates. The average PACU stay was similar in both groups at around 4.0 hours (p = 0.0770). However, the length of hospital stay was significantly longer in the DIE group (p < 0.001). Time to ambulation was significantly longer in the DIE group (p < 0.001), whereas time to return to work did not differ significantly between the two subgroups. Table 5 presents the changes in pain and QoL scores from the preoperative to postoperative period.

**Table 5:** Changes in pain and QoL scores from the preoperative to postoperative period. *: Indicates there is statistical significance between the two timepoints; ^#^: p-value calculated using a paired sample t-test. DIE: deep infiltrating endometriosis; SD: standard deviation; VAS: Visual Analog Scale; QoL: quality of life; EHP-30: Endometriosis Health Profile questionnaire

Parameters	Preoperative	Postoperative period	% change	t-score/chi-square	P-value
Non-DIE subgroup (N = 65)					
Pain score (VAS scale), mean ± SD^#^	7.5 ± 0.9	1.9 ± 0.9 (24 hours)	-74.7	35.1152	<0.001*
		0.1 ± 0.4 (7 days)	-98.7	64.0000	<0.001*
QoL (EHP-30), mean ± SD^#^	75.8 ± 7.5	26.7 ± 5.8 (7 days)	-64.8	39.5837	<0.001*
DIE subgroup (N = 43)					
Pain score (VAS scale), mean ± SD^#^	8.4 ± 1.2	2.3 ± 0.6 (24 hours)	-72.6	29.0174	<0.001*
		0.8 ± 0.9 (7 days)	-90.5	49.7022	<0.001*
QoL (EHP-30), mean ± SD^#^	82.2 ± 8.0	33.5 ± 8.8 (7 days)	-59.2	40.9646	<0.001*

Both the non-DIE and the DIE subgroups showed significant improvements in pain and QoL scores postoperatively compared to their preoperative values (p < 0.001).

Learning curves

**Figure 1:**
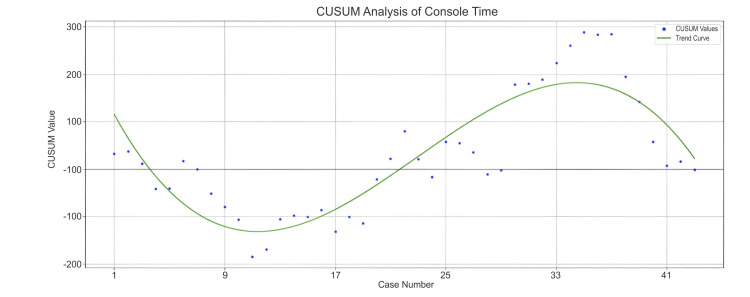
CUSUM curve of operative time (DIE cases). CUSUM: cumulative sum; DIE: deep infiltrating endometriosis

We evaluated the learning curve for DIE cases using a CUSUM analysis of operative time (Figure 1), which revealed three distinct phases.

Phase 1 (Cases 1-20) showed initial fluctuations followed by a downward trend, with the curve dipping below zero, indicating improving efficiency and a rapid learning phase that transitioned into a stable period of proficiency. Phase 2 (Cases 20-36) demonstrated a sharp and sustained upward trend, with the curve rising steeply and peaking around Case 35. This phase corresponded with a shift in case selection, involving increasingly complex cases. Phase 3 (Cases 36-44) reflected a marked improvement, as the curve declined rapidly from its peak and ended slightly below zero, signifying a recovery in performance and a return to baseline operative efficiency.

**Figure 2:**
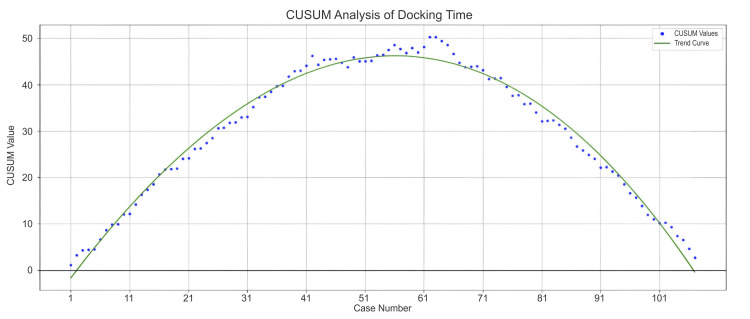
CUSUM curve of docking time for the overall population. CUSUM: cumulative sum

**Figure 3:**
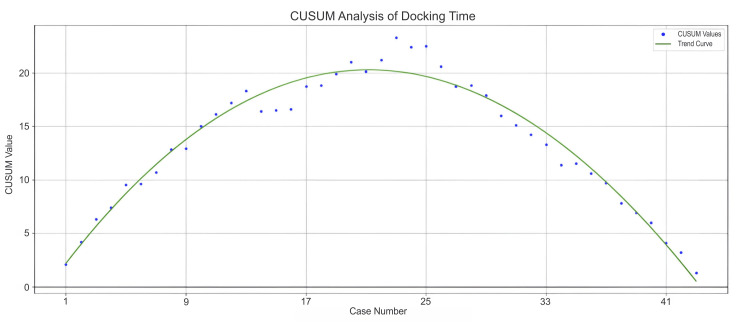
CUSUM curve of docking time for the DIE subgroup. CUSUM: cumulative sum; DIE: deep infiltrating endometriosis

In addition to operative time, we also analyzed trends in docking times, which serve as an important indicator of operating room staff efficiency. Figure 2 and Figure 3 depict the CUSUM learning curves for docking times in the overall population and the DIE subgroup, respectively.

Both curves exhibited a characteristic learning curve pattern, reflecting initial variability followed by a period of progressive improvement and stabilization in docking efficiency. For the overall population, Phase 1 (Cases 1-42) exhibited a steady and prolonged upward trend, indicating consistently longer docking times than the target, which reflected a clear learning curve or increasing case complexity. Phase 2 (Cases 43-62) was characterized by fluctuations, with an initial improvement followed by a temporary setback. The CUSUM values declined modestly before rising again, suggesting early adaptation followed by procedural variability or the presence of challenging cases. Phase 3 (Cases 63 onwards) demonstrated a sustained and significant improvement, with the trend steadily declining, crossing the baseline, and approaching zero, signalling increasing proficiency, streamlined workflow, and consistent efficiency gains over time.

Similarly, for the DIE subgroup, the CUSUM trend also had three classical phases for docking time. In Phase 1 (Cases 1-23), the curve showed a steady upward slope, indicating consistently longer docking times than the target. This suggested a learning phase marked by initial complexity. Phase 2 (Cases 23-28) was characterized by minor fluctuations, with the curve dipping slightly and then stabilizing. This represented a transitional period where performance began to stabilize but remained above the target. In Phase 3 (Cases 28-43), the curve showed a clear and sustained downward trend. This reflected a significant improvement in docking efficiency, as the team gained proficiency and approached the target performance.

## Discussion

Endometriosis affects 6-10% of women of reproductive age, with a prevalence of 38% (20-50%) in infertile women and 71-87% in those with chronic pelvic pain [4]. Surgical resection of DIE significantly improves pain and QoL [8]. MIS is the gold standard for treatment [8], with laparoscopy preferred over laparotomy due to comparable long-term outcomes [7]. Although RAS is considered an advancement within MIS [9], concerns regarding cost and limited high-quality evidence persist, warranting further investigation. RAS originated from NASA and U.S. military-funded research in the 1970s, leading to the development of the first da Vinci Surgical Systems by Intuitive Surgical in the early 2000s [9]. Initially used in gynecologic oncology, RAS has recently been adopted for endometriosis treatment, including DIE, with studies supporting its safety and feasibility [12-15]. Current evidence suggests RAS is non-inferior to laparoscopy, offering benefits such as reduced pain, shorter hospital stays, and fewer complications [9,16]. The role of RAS in endometriosis remains a topic of ongoing debate, balancing its advantages, such as enhanced precision, visualization, and optimization of MIS, with limitations such as high cost [17]. Its limited use is often due to expensive acquisition and maintenance, restricted access in multi-specialty centers prioritizing oncology, and the need for trained surgeons [17]. Despite limited studies, evidence over the past decade supports the feasibility of RAS for DIE [17,18], and French guidelines (2018) endorse it as a frontline option for DIE with digestive involvement [19]. This study evaluated the short-term outcomes of RAS across various stages of endometriosis in the Indian context.

Baseline and preoperative variables of the study population

The preoperative characteristics of our patient subgroups, including age and BMI, align with global and Indian data [20,21]. The mean age was 40.9 ± 6.6 years and mean BMI 26.3 ± 3.3 kg/m², comparable to a prior randomized controlled trial reporting 34.3 ± 7.2 years and 26.1 ± 5.2 kg/m² [21], and an Indian study showing 34.5 ± 3.5 years and 24.9 ± 3.8 kg/m², respectively, in the robotic group [20]. In our study, 25.9% had infertility, similar to rates in earlier Indian studies (35.7% [20], 45.7% [21]). Most patients (60.2%) had Stage I-II disease, while 39.8% had Stage III-IV (DIE), consistent with a multicentric study reporting more early-stage cases in the robotic group [21]. In this study, the non-DIE subgroup had a preoperative mean pain score of 7.5 ± 0.9 on the VAS scale. A previous study reported that 77.1% of patients undergoing robotic surgery for endometriosis cited pelvic pain as the primary indication [21]. Another study identified dysmenorrhea as the most painful symptom (mean VAS: 5.76), followed by dyspareunia (2.87), chronic pelvic pain (2.55), dysuria (1.19), and dysuria (0.19) [22]. The mean preoperative QoL, assessed by the EHP-30 score, was 75.8 ± 7.5 in our non-DIE subgroup.

Perioperative outcomes of the study population

Intraoperative and postoperative outcomes are key measures of surgical safety. In this study, the mean operative time and docking time for RAS were 129.8 ± 56.0 and 5.9 ± 1.3 minutes, respectively. These findings are comparable to the American LAROSE study, which reported an operative time of 106.6 ± 48.4 minutes and a docking time of 4.6 ± 5.9 minutes (p = 0.71) [21], and an Indian study reporting a mean operating time of 100.3 ± 39.9 minutes for RAS [20]. In 90.7% of cases, only a gynecologist was involved, while 9.3% required a multidisciplinary team (gynecologist with urologist or colorectal surgeon), consistent with prior evidence that surgery for DIE often demands complex, multidisciplinary approaches due to higher complication risks [23]. Firefly™ technology was utilized in 12.1% of patients. A previous study demonstrated that robotic single-incision laparoscopy with Firefly and indocyanine green enhances visualization and facilitates the removal of otherwise invisible endometriotic lesions [24].

The mean estimated blood loss in this study was 53.0 ± 47.3 mL, lower than that reported in a previous Indian study (78.5 ± 46.8 mL) [20] and a multicentric study (100.9 ± 229.8 mL) [21]. Blood transfusion was required in 3.7% of patients, aligning with data from a multi-institutional Indian study, which reported a lower transfusion requirement in benign cases (0.02 units of packed red blood cells) compared to malignant cases (0.05 units) [10]. Intraoperative complications occurred in 0.9% of patients, with one patient also experiencing a postoperative complication; no wound infections were reported. These findings are favorable compared to previous data, where one (2.9%) patient had a small bowel injury intraoperatively, and postoperative complications included wound infection (8.6%), urinary tract infection (8.6%), and vaginal bleeding (one case) [21]. Notably, no conversions to open surgery were required in our subgroups, consistent with international findings reporting zero conversions [21].

In this study, the mean hospital stay was 2.2 ± 0.7 days, and the mean PACU stay was 4.0 ± 0.3 hours. These findings align with a multi-institutional Indian study reporting a mean length of stay of 2.07 days for benign cases [10]. The same study assessing robotic surgery for benign conditions across two time periods reported a mean length of stay of 2.12 days (2011-2015) and 2.23 days (2016-2021) [10]. Notably, no patient in our subgroup required reoperation, and the mean time to return to work or resume full household activities was 2.2 ± 0.9 days.

Subgroup analysis: deep infiltrating endometriosis cases

The preoperative characteristics of patients in the DIE subgroup were consistent with global data [17,25]. The mean age was 36.4 ± 6.2 years, and the mean BMI was 26.2 ± 3.1 kg/m². These values are comparable to a retrospective French multicentric study (2008-2019) from the Society of European Robotic Gynecological Surgery endometriosis database, which reported a median age of 34 years (interquartile range (IQR): 30-40) and a mean BMI of 22.9 kg/m² (IQR: 20.9-26.5) [17]. In the DIE subgroup of this study, the preoperative mean pain score (VAS) was 8.4 ± 1.2, indicating a high symptom burden. This aligns with findings from a French multicentric study (2008-2019), where 76.8% (238/310) of DIE patients reported chronic pelvic pain [17]. The mean preoperative QoL score, assessed by EHP-30, was 82.2 ± 8.0 in our subgroup. Notably, infertility was present in 51.2% of DIE cases, higher than the 34.1% (107/314) reported in the same French study, which also documented a median infertility duration of two years (IQR: 1.25-4.0) [17].

Non-deep infiltrating endometriosis versus deep infiltrating endometriosis subgroup

In this study, perioperative and functional outcomes were compared between the non-DIE subgroup (n = 65) and the DIE subgroup (n = 43). The operative time was significantly longer in DIE cases (161.4 ± 51.2 vs. 108.8 ± 49.5 minutes, p < 0.001). This finding aligns with a retrospective French multicentric study (2008-2019), which reported a high median operative time of 245 minutes (IQR: 186-320) and an average console time of 138 ± 75 minutes; the average docking time was 54 ± 18 minutes [17]. Similarly, a multicenter study showed that, after adjusting for surgery type, operative time for patients with Stage I/II or no endometriosis was 76 minutes shorter than for those with Stage III/IV disease (p < 0.001) [21]. As expected, milder disease stages were associated with shorter operative times compared to advanced endometriosis [21]. A significantly higher proportion of patients in the DIE subgroup required blood transfusion (p = 0.023), whereas no patients in the non-DIE subgroup required transfusion. Previous studies have reported the following findings: one reported a mean blood loss of 70 ± 107 mL (median: 50 mL; IQR: 0-100) during robotic endometriosis surgery, with a perioperative transfusion rate of 0.6% [17], while another study reported an estimated blood loss of 100 mL (IQR: 50-200) in DIE patients [25]. The length of hospital stay was longer in the DIE group compared to the non-DIE subgroup (2.6 ± 0.7 vs. 2.0 ± 0.6 days, p < 0.001). This aligns with a previous study reporting a median hospital stay of four days (IQR: 3-6) and an average duration of five days for DIE patients undergoing robotic surgery [17]. The mean duration of PACU stay was comparable between the two groups (approximately 4.0 hours; p = 0.077).

Pain and quality of life scores

Both the non-DIE and the DIE subgroups showed significant postoperative improvements in pain and QoL scores compared to their preoperative values (p < 0.001). These findings are consistent with a previous study that reported significant enhancements across all QoL domains, i.e., pain, control/powerlessness, emotions, social support, self-image, work, children, sexual intercourse, medical care, and treatment, at both six weeks and six months post-surgery [21].

Learning curves

To our knowledge, this is one among a handful of studies that have analyzed the learning curve for DIE. Considering the complexity of DIE cases, compared to non-DIE cases, we restricted our analysis to DIE.

In our analysis, the CUSUM curve for total operative time showed a marked downward trend, indicating significant performance improvement. The curve declined sharply from its peak and crossed below zero between Cases 36 and 44, suggesting the surgeon had achieved or exceeded the expected efficiency with minimal variability. Similarly, the CUSUM curves for docking time, both in the overall population and the DIE subgroup, exhibited a steady and sustained decline. The trend crossed the baseline and approached zero around Case 63, reflecting growing proficiency, streamlined workflow, and consistent efficiency improvements over time. A multi-institutional Indian study reported a significant reduction in mean console time for benign cases over the last five years (p = 0.02), with times decreasing from 99.18 minutes (2011-2015) to 70.56 minutes (2016-2021) [10]. The study also noted a marked decline in docking time, from 26.75 minutes in the initial five years (n = 476) to 14.70 minutes in the subsequent five years (n = 1065) [10]. In line with this, the CUSUM analysis for docking time in the DIE subgroup revealed a sustained downward trend, indicating continuous improvement in docking efficiency. The curve approached the performance baseline between Cases 28 and 43, reflecting growing team proficiency and increasingly streamlined robotic setup.

Limitations and future directions

This study has several limitations that merit consideration. Its retrospective design may introduce selection bias and limit causal inference. Being a single-center study conducted by a single experienced surgeon, the findings may not be fully generalizable to centers with lower procedural volumes or surgeons earlier in their learning curve. Additionally, although significant improvements in pain and QoL were observed, long-term outcomes, including disease recurrence, fertility outcomes, and durability of symptom relief, were not evaluated. The absence of a laparoscopic comparator group and lack of cost or cost-effectiveness analyses, particularly relevant in the Indian context, are additional limitations. Future research should involve prospective, multicenter comparative studies with standardized outcomes, long-term follow-up, and health-economic evaluations to better define the role of RAS for endometriosis.

## Conclusions

This retrospective, non-comparative analysis presents our preliminary institutional experience with RAS for endometriosis across varying disease stages in the Indian setting. Both non-DIE and DIE subgroups demonstrated meaningful postoperative improvements in pain and QoL, with low complication rates and no conversions to open surgery. Although DIE cases were associated with longer operative durations, perioperative outcomes remained favorable and aligned with published international benchmarks. CUSUM-based learning curve analysis demonstrated progressive improvements in operative and docking efficiency, indicating increasing surgical proficiency and optimization of operating room workflows over time. Nevertheless, given the limitations of the study design, definitive conclusions regarding safety or comparative effectiveness cannot be established. Well-designed, adequately powered prospective comparative studies are required to more clearly define the clinical role of RAS in endometriosis management.
